# Study on the alteration of gut microbiota in ovariectomized rats and the impact of estrogen intervention over a 3-month period

**DOI:** 10.1371/journal.pone.0330108

**Published:** 2025-09-04

**Authors:** XinDong Lei, TingTing Cheng, Han Dong, JieYing Xia, Yang Hong, GuoQiang Cheng, YongJin Wang, TieZhu Chen

**Affiliations:** Animal Experiment Center, Sichuan Academy of Chinese Medicine Sciences, ChengDu, SiChuan, China; Universidade do Estado do Rio de Janeiro, BRAZIL

## Abstract

Postmenopausal osteoporosis (PMOP) is a common primary osteoporosis. With the aging of the population, it is becoming a major disease that endangers health and quality of life. The purpose of this study was to explore the effect of gut microbiota on PMOP by observing the changes in the levels of estradiol, bone density, and gut microbiota diversity in rats after 3 months of OVX surgery. 60 female SD rats were randomly divided into four groups: baseline group (6 rats), sham-operated group (18 rats), model group (18 rats), and estrogen-treated group (18 rats). The ovariectomy model of postmenopausal osteoporosis was established by performing bilateral ovariectomy. After surgery, 6 rats from each group were randomly selected for sacrifice every 30 days and subsequent assessment. At the end of 90 days, all rats were sacrificed for evaluation of body weight, bone mineral density (BMD), tissue mineral density (TMD), trabecular bone parameters, femoral bone morphology, hormone levels, and gut microbiota diversity. The analysis revealed that OVX led to a decrease in BMD, TMD, and serum estradiol levels in rats, and increased TNF-α levels. The bone micro-architecture and tissue morphology were also changed, with trabecular fractures, thinning, and decreased numbers. Meanwhile, there was also a shift in the diversity of gut microbiota. The administration of estrogen could potentially ameliorate these alterations. Overall, OVX leads to a persistent decline in estrogen levels in rats. This results in gradual bone loss, which is related to gut microbiota imbalance.

## 1 Introduction

Osteoporosis is a systemic metabolic bone disorder characterized by reduced bone mass and deterioration of bone microarchitecture, resulting in increased bone fragility and susceptibility to fractures, this condition is predominantly observed in postmenopausal women due to estrogen deficiency [1]. As the global population ages, postmenopausal osteoporosis is becoming a major public health problem, with a significant impact on health and economic costs [2,3].

Postmenopausal estrogen deficiency is the leading cause of postmenopausal osteoporosis (PMOP). Estrogen directly impacts osteoblasts, osteoclasts, and osteocytes, which are the three types of cells composing bone tissue. Estrogen deficiency may lead to an increase in bone resorption and a decrease in bone formation, resulting in alterations in bone microstructure and subsequent bone loss [4,5]. The interaction between immune cells, inflammatory factors, and bone metabolism is an important part of the pathogenesis of osteoporosis [6,7]. In postmenopausal women, circulating T-cell levels are usually normal or elevated, which can lead to enhanced expression of tumor necrosis factor-alpha (TNF-ɑ), induction of osteoblast apoptosis, and indirect stimulation of osteoclastogenesis through the Receptor Activator of Nuclear Factor-κ B Ligand (RANKL) pathway, and these changes can cause bone loss [8,9].

Gut microbiota(GM), the general term for microorganisms that colonize the human gut, plays an important role in the pathogenesis of PMOP [10]. The gut microbiome of postmenopausal women has been found to undergo significant changes that are closely linked to BMD and clinical outcomes [11]. GM can interfere with bone metabolism and affect bone health through various pathways such as the immune system, intestinal calcium absorption, and neurotransmitter release [11–13]. For example, GM may optimize the longitudinal growth of the skeleton, including femur length, cortical thickness, and the trabecular fraction of the femur, by improving growth hormone sensitivity and consequently increasing IGF-1 activity in bone^[14]^. Animal research^[15]^ demonstrated that germ-free mice showed increased trabecular and cortical bone mass compared to normal mice, as well as reduced expression of inflammatory factors in the bone marrow. These differences were restored to normal levels after receiving gut microbiota transplantation from normal mice. Additionally, it was noted that germ-free mice exhibited resistance to bone loss induced by estrogen deficiency [14]. Obviously, GM plays an important role in bone loss caused by estrogen deficiency.

However, the development of postmenopausal osteoporosis is a long-term and gradual process, during which the dynamics of estrogen, inflammatory factors, and gut microbiota remain unclear^[17]^. Previous studies have mainly focused on changes in animals after successful modeling, with little exploration of the evolving trends and correlation between gut microbes and bone during the modeling process. Therefore, this study employed experimental methods including 16S rRNA sequencing and statistical analysis to investigate the dynamic changes of various indicators within 3 months following ovariectomy in rats. The primary objective was to explore the population diversity changes and underlying mechanisms of intestinal flora during the development of osteoporosis induced by estrogen deficiency. These findings will contribute to a better understanding of the pathogenesis of postmenopausal osteoporosis and facilitate the development of novel treatments for this condition.

## 2 Methods

### 2.1 animals

Sixty SPF-grade female Sprague-Dawley (SD) rats, 6 months old, weighing 337.98 + 33.31 g, were provided by the Animal Experiment Center of Sichuan Academy of Chinese Medicine Sciences(SACMS). The rats were housed in SPF-grade barriers in the Animal Experiment Center of SACMS. The temperature was maintained at 22 ± 3°C and the light/dark cycle was 12 hours. All animals had free access to water and standard food. All procedures were reviewed and approved by the Experimental Animal Ethics Committee of SACMS (acceptance number R20220301-1). The procedures were implemented in accordance with the requirements of the Committee.

### 2.2 Animal model establishment and treatment

After adaptive feeding week, the 60 rats were randomized into four groups: the 6 – rat baseline group (JX), and the model (M), sham – operated (J), and estrogen treatment (C) groups, each with 18 rats. These three groups were further split into three 6 – rat subgroups: first – month (A), second – month (B), and third – month (C). For example, the model group has subgroups MA, MB, and MC. Both the model and estrogen treatment groups underwent bilateral ovariectomy. Briefly, following anesthesia induction and prophylactic antibiotic administration, a midline abdominal incision was made at the level of the bilateral inguinal regions in rats. Both ovaries were excised via this approach, and the vascular pedicle was ligated using a 3−0 surgical suture, followed by layered closure of the abdominal wall. The rats in the sham-operated group were subjected to abdominal incision at the same position to expose the bilateral ovaries, only a piece of adipose tissue of similar size near the ovaries was excised. The rats were housed in pairs and received daily intramuscular injections of sodium penicillin post-surgery to prevent infection. After three days of continuous monitoring, it was confirmed that the rats showed no apparent abnormal conditions, and then normal feeding was resumed.

In order to gather data on the initial state, rats in the JX group were conventionally fed without any intervention. One week after operation, the rats in the estrogen treatment group were treated with estradiol valerate (PROGYNOVA, Bayer)by gavage administration, and the rats in the sham-operated group and the model group were given the same volume of normal saline. The dosage of E2 (50 μg/kg/ d) was meticulously determined based on the equivalent dose ratio derived from converting the human body surface area to that of rats. In addition, relevant literature was thoroughly consulted to ensure the rationality and scientific validity of the dosage determination [15]. The administration was continued for 3 months. During the treatment period, the rats were kept awake and as comfortable and safe as possible.

**Fig 1:**
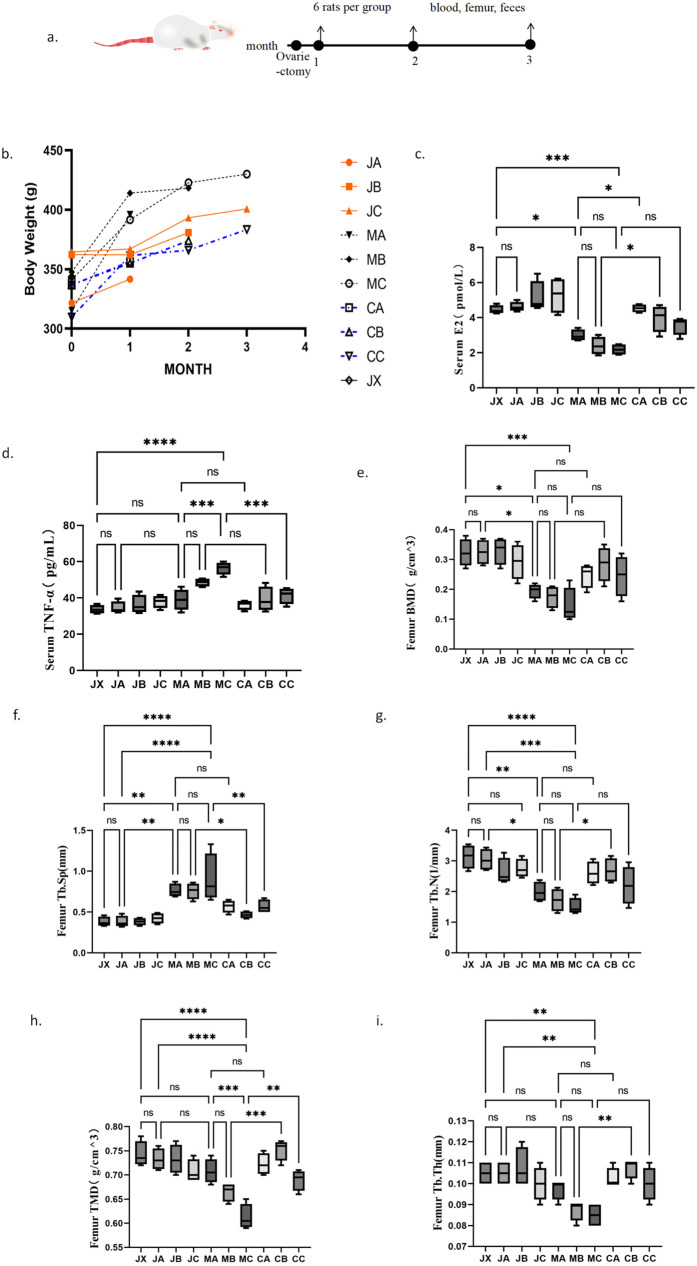
Experimental design,trend of the body weight changebody weight, serum E2, TNF-α, and Bone tissue metrology results. **(a)** Experimental design, refer to Section 2.2 in the Methods section. **(b)** The trend of body weight changes in each group of rats. (c-d) serum levels of estrogen and TNF-ɑ. (e-i) Quantitative analysis of BMD, TMD, Tb. N, Tb. Th, and Tb. Sp.*P < 0.05, **P < 0.01.****P < 0.0001.

After 1 – week acclimatization, JX group rats were overnight – fasted, weighed, and anesthetized with 50 mg/kg sodium pentobarbital (2% solution) via intraperitoneal injection. Blood was collected from the abdominal aorta, centrifuged at 4°C and 3000 rpm for 10 min, and the supernatant stored at −80°C. The left femur, with peeled – off soft tissue, was wrapped in saline – moistened gauze and stored at −80°C for histopathological testing. Feces were collected via stress defecation, packed into sterile EP tubes, sealed, stored at −80°C, and transported on dry ice. The remaining rats were sacrificed similarly after the feeding period, with samples collected and processed as above (Fig 1a for experimental design).

### 2.3 Biochemical analysis

After collecting blood from the abdominal aorta, the obtained whole blood was centrifuged at 3000 rpm for 10 minutes at 4°C. The supernatant was then collected and stored in EP tubes labeled with corresponding numbers. The samples were stored in a −80°C refrigerator and should be thawed before use. Serum estradiol (E2) and inflammatory immune factor TNF-α levels were determined by ELISA using corresponding kits (Shanghai Zhuocai Biotechnology Co., Ltd.), according to the manufacturer’s instructions.

### 2.4 Micro-CT scanning and analysis

Calibrated femurs were fixed in 10% neutral buffered formalin for 24 hours, stripped of excess soft tissue, and scanned via a Skyscan 1276 micro – CT instrument (Bruker microCT, Kontich, Belgium) at 55 kV source voltage, 200 μA source current, with an AI 0.25 mm filter and 6 μm pixel size, and a 0.3 – degree rotation step. Use NRecon software (Bruker microCT, Kontich, Belgium) to reconstructed images, with ring artifact correction at 5, smoothing at 3, and beam hardening correction at 30%. CTAn program (Bruker microCT, Kontich, Belgium) analyzed trabecular bone parameters (Tb. N, Tb. Th, Tb. Sp, BMD, TMD) using the same source voltage, current, filter, pixel size, and rotation step as the scan. A refined volume of interest was defined as 1 mm above the growth plate of the distal femur and 3 mm in height. Within this volume, the trabecular bone ROI was manually delineated, and bone parameters within it were determined using a constant 80–255 threshold for trabecular bone binarization.

### 2.5 Hematoxylin-eosin staining

After fixation, the tissue was immersed in a 15% EDTA solution for decalcification, with fresh solution replacement every3 days until decalcification completion. Decalcification endpoint was confirmed when an acupuncture needle could easily penetrate the bone tissue. Subsequently, the tissue underwent dehydration in an automatic processor with the following schedule: 75% alcohol for 30 h, 85% alcohol for 4 h, 95% alcohol for 8 h, 100% alcohol for 7 h (in four intervals: 2 h, 2 h, 1 h, and 1 h), followed by two xylene baths for 45 min each, and three paraffin baths for 5 h each. The dehydrated tissue was embedded in paraffin, sectioned at 5 mm thickness, and with stained HE following SOPs. A Panoramic 250 scanner (3DHISTECH, Hungary) was used to digitize the sections. Initial low-magnification observation assessed overall tissue lesions, after which specific areas were selected for 40x and 100x imaging to examine detailed lesions. Evaluation focused on the microstructural changes of sliced tissues, including bone trabeculae area, structure, and shape. Histological assessment was performed by Zhendong Zhong, a Chinese veterinary pathologist.

### 2.6 Gut Microbiota biodiversity analysis

The frozen fecal samples were thawed at −4°C and genomic DNA was extracted using the Soil and Feces Genomic DNA Extraction Kit with magnetic beads (TIANGEN, Beijing, China; Soil and Feces Genomic DNA Extraction Kit; catalog number: DP712). PCR reactions consisted of 15 µL Phusion® High-Fidelity PCR Master Mix, 0.2 µM primers, and 10 ng genomic DNA. PCR conditions were 98°C for 1 min, followed by 30 cycles of 98°C for 10 s, 50°C for 30 s, and 72 for 30 s, with a final extension at 72°C for 5 min. PCR products were visualized via electrophoresis on a 2% agarose gel.

The NEB Next® Ultra™ II FS DNA PCR-free Library Prep Kit (NEB/E7430L) was used for library construction. Use FLASH after truncating the Barcode and primer sequences [16], the reads of each sample are spliced to get the Raw Tags. Use fastp software to process the spliced Raw Tags through strict filtering to obtain high-quality Tags data [17]. The Tags sequence is compared with the species annotation database (Silva database) to detect the chimera sequence, and finally remove the chimera sequence to obtain the Effective Tags [18]. Then use the DADA2 module in the QIIME2 (Version QIIME2–202006) software to perform noise reduction to obtain the final ASVs (Amplicon Sequence Variants) and feature table [19]. The species annotation was conducted using QIIME2 software with the Silva 138.1 database. Subsequently, the data from each sample was standardized based on the sample with the lowest amount of data. For diversity analyses, standardized data were used. Beta diversity was assessed in QIIME2 via weighted and unweighted Unifrac distances. Cluster analysis involved PCA, with three – dimensional PCoA results from QIIME2 and two – dimensional results from R’ s ade4 and ggplot2 packages (version 4.0.3). LEfSe analysis was performed with an LDA score threshold of 4. MetaStat analysis, via R software, evaluated differences across six taxonomic levels. Species with a *p*-value less than 0.05 were identified as significantly different between groups.

### 2.7 Statistical test

Data are presented as mean ± standard deviation (SD). One-way analysis of variance (ANOVA) was used to compare data between groups. Post hoc comparisons were performed using the Tukey-Kramer method. Statistical significance was set at P < 0.05, and significant differences were denoted by *P < 0.05 and **P < 0.01. SPSS statistical software (version 25.0; IBM Corporation, Armonk, NY, USA) was used for all analyses.

## 3 Results

### 3.1 OVX osteoporotic rats serum E2, TNF-α, bone mineral density, bone mineral content, bone trabecular structure, bone microstructure changes and the influence of estrogen on them

During the experimental feeding period, the body weight of all rats exhibited a significant increase, with the specific trend illustrated in Fig 1b. The systemic estrogen level of the rats was significantly lower in the model group than in the sham-operated group. The serum estradiol level of the rats in the model group gradually decreased over time and was significantly different from the level in the first month. Estrogen treatment significantly restored the serum estradiol levels of the rats in the model group (Fig 1c).

We also found that the level of TNF-α in the serum of rats in the model group gradually increased over time, and was significantly different from the level in the sham-operated group from the second month (Fig 1d). These results suggest that ovariectomy (OVX) causes a gradual decrease in systemic estrogen levels and a gradual increase in TNF-α levels in rats, which can be effectively improved by estrogen treatment.

Micro-CT was used to observe the changes in bone loss in ovariectomized (OVX) rats and the therapeutic effect of estrogen on it. Bone mineral density (BMD), trabecular mean density (TMD), trabecular number (Tb.N), and trabecular thickness (Tb.Th) of rats in the model group decreased progressively over time, while trabecular separation (Tb.Sp) increased progressively (Fig 1h-1i). Compared to the model group, the estrogen treatment group had significantly higher BMD, TMD, Tb. N, Tb. Th, and lower Tb.Sp, suggesting that estrogen treatment can reverse OVX-induced bone loss and improve osteoporosis symptoms.

**Fig 2:**
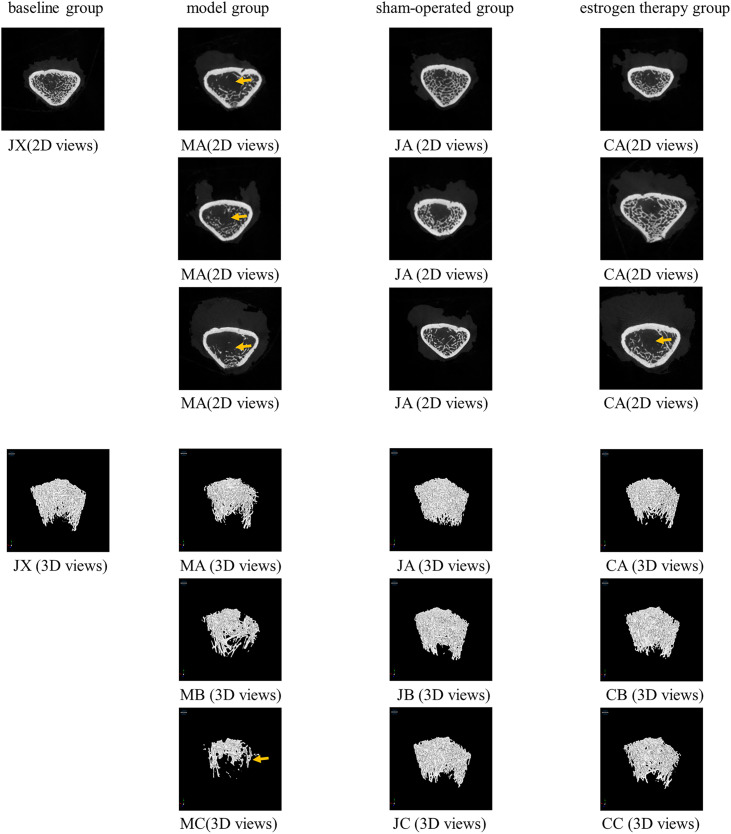
Representative samples from each group showing 2D mapping and 3D structural models of trabecular bone within the metaphyseal region of the distal femur. The group information is located at the top of the picture, and the 2D or 3D picture annotations are located below each picture.thinner bone regions (↑).

Representative 2D and 3D micro-CT images of rat femurs in each group are shown in Fig 2. The images show that rats in the model group had significant bone loss and bone structure deterioration compared to the sham-operated group. The femoral head of rats in the model group was more porous, and the bone trabeculae were thinner and more fractured. Estrogen treatment significantly improved the bone structure damage caused by OVX.

### 3.2 Morphological changes in bone tissue of OVX osteoporotic rats and effects of estrogen

**Fig 3:**
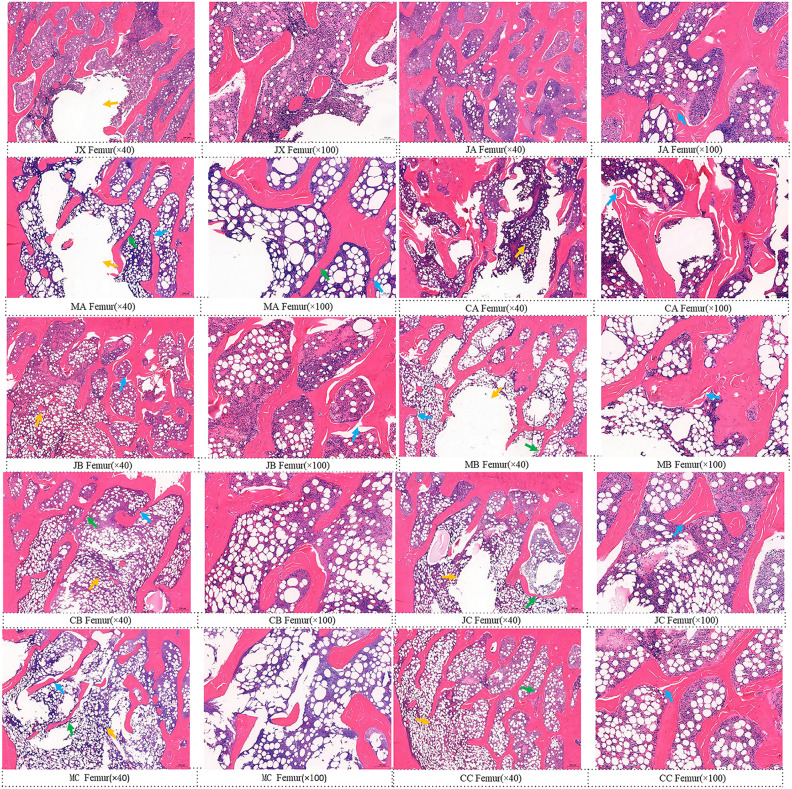
Representative images of tissue sections of the metaphyseal region of the distal femur stained with hematoxylin-eosin (HE). The magnification of the optical microscope is displayed beneath the image. The notable areas are indicated by three colored arrows, specifically: trabecular thinning(↑) trabecular fracture(↑) and Decreased number of trabecular(↑).

The distal femoral metaphysis of rats in each group was stained with HE, and representative histological images of each group were selected (Fig 3). Compared with the baseline group and the sham-operated group, the femur tissue of the rats in the model group showed obvious morphological changes, including bone trabecular thinning, fracture, and number reduction, and obvious osteoporotic changes. These changes became more severe with time in all groups. However, the sham-operated group and the estrogen group had less bone loss than the model group, with relatively more and thicker bone trabeculae. These results suggest that ovariectomized (OVX) rats experience progressive bone loss over time, and that estrogen treatment can delay this change and improve bone microarchitecture.

### 3.3 Change trend of gut microbiota diversity in ovariectomized (OVX) osteoporotic rats and the effect of estrogen on it

To explore the trend of intestinal microbial changes in ovariectomized (OVX) osteoporotic rats, we performed 16S rRNA sequencing to analyze the composition of intestinal microbial taxa after OVX.

**Fig 4:**
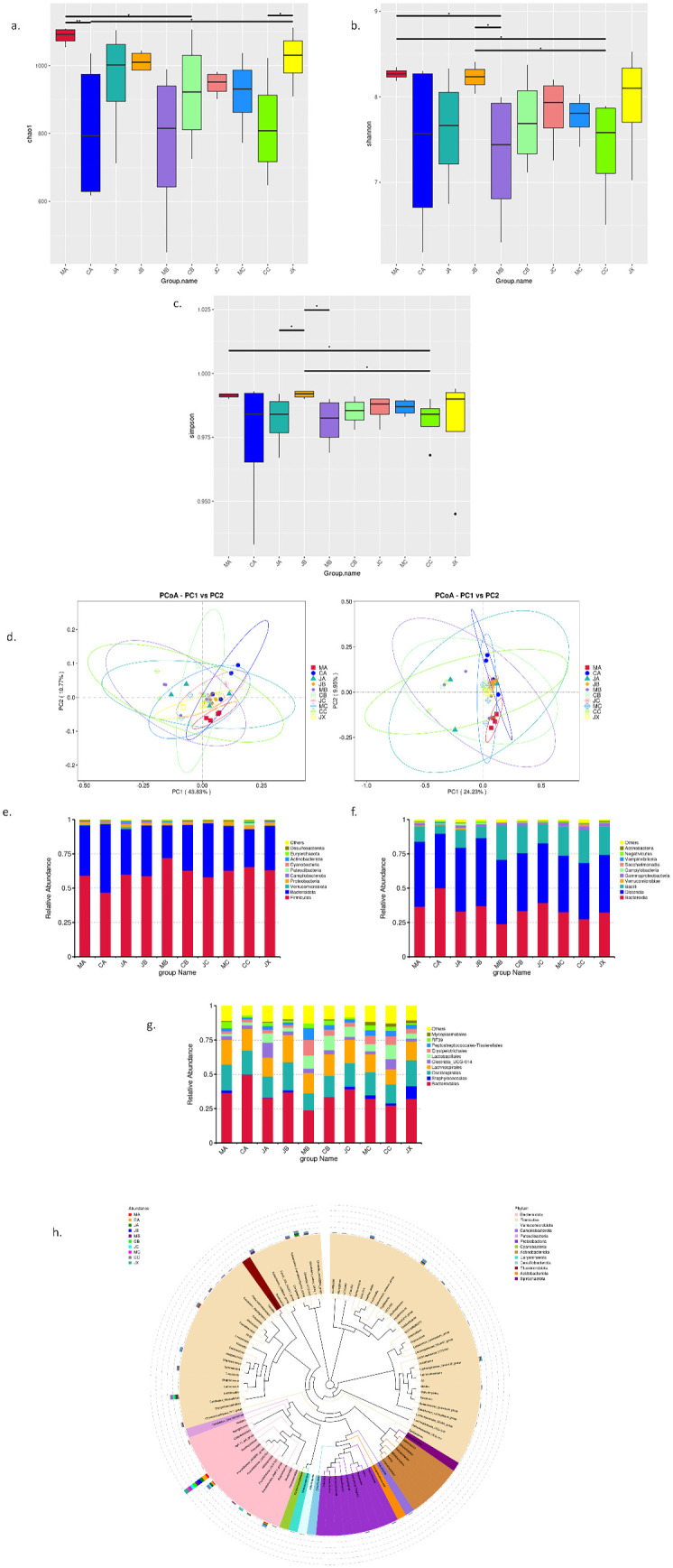
Gut microbial diversity measurements in ovariectomized rats. **(a-c)** The Alpha test results, Chao1, Shannon and simpson are located in the three panel a, b and c respectively. **(d)** The PoCA results of Beta detection. **(e-g)** The differences in biodiversity at the three levels of phylum **(e)**, class **(f)**, and order **(g)**. **(h)**The species phylogenetic tree of the Top100 species. Statistical differences are indicated by * above the boxplots, *P < 0.05.

Alpha diversity: In the second month, the Chao1 and Shannon indexes were significantly different between the model group and the sham-operated group, but there was no significant difference between the estrogen treatment group and the sham-operated group (Fig 4a–4c). This suggests that OVX may have a significant effect on the species richness of the microbiota, which may be ameliorated by estrogen treatment. However, this difference was not reflected in the third month or the first month, and the Simpson index did not reflect a significant difference.

Beta diversity: To detect the degree of similarity among different microbiota, we also analyzed β-diversity using PCoA and NMDS. The results (shown in Fig 4d) showed that the microbiota of each group showed obvious clustering, indicating that the model rats were significantly different from the sham-operated group and the estrogen treatment group, and this difference gradually increased over time.

Then, to assess specific changes in microbial species, we further analyzed the relative abundance of major taxa. At the phylum level, there were differential changes in Campylobacter, Actinomycetes, Cyanobacteria, Firmicutes, and Bacteroides in the first month and second months in the model group. However, by the third month, this difference was not obvious. At the class level, the number of bacillus changes significantly. At the order level, Bacteroides, Oscillating Spirilles, Lachnospira, Clostridia_UCG-014, and Lactobacillus are the largest bacterial orders. In addition, in order to further study the phylogenetic relationship of species at the genus level, the representative sequences of the top 100 genera were obtained through multiple sequence alignment, and are shown in Fig 4e, 4f, 4g, 4h.

**Fig 5:**
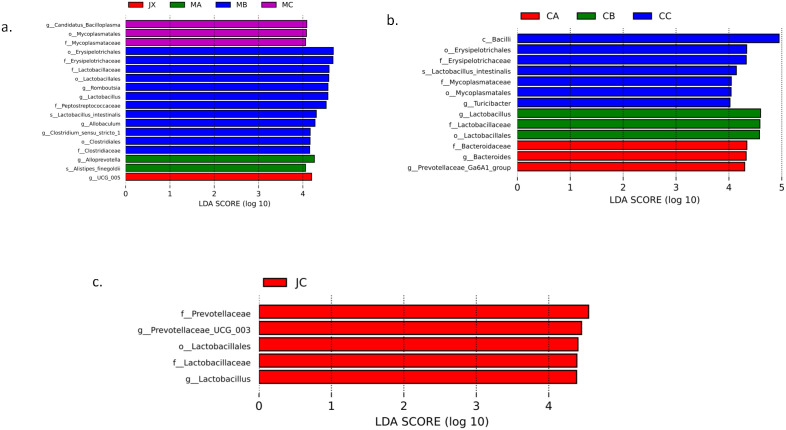
The longitudinal changes in the intestinal microbiota of the model group rats over three months of modeling. (a) The overall changes in the model group over time. **(b)** Major species that changed over three months in the estrogen-treated group. **(c)** The major species that changed over three months in the sham group.

Using the LEfSe tool, we analyzed the changes in intestinal microorganisms in the model group rats within three months of modeling. We found that at the order level, Erysipelotrichales, Lactobacillales, and Clostridiales all had significant changes in the second month. Mycoplasmatales had significant changes in the third month. Notably, this longitudinal difference was relatively small in both the sham and estrogen-treated groups. These findings once again demonstrate that OVX induces gut microbial imbalance in rats and that estrogen treatment can improve this condition to some extent. The results are shown in Fig 5a, 5b, 5c.

**Fig 6:**
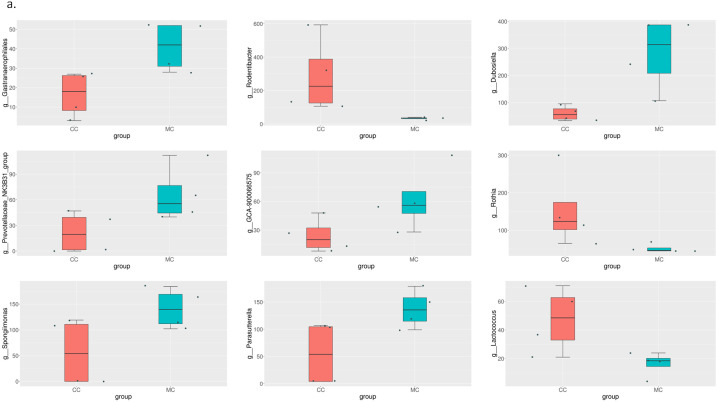
MetaStat analysis results at the order classification level. Differences between the MC group and CC group.

**Fig 7:**
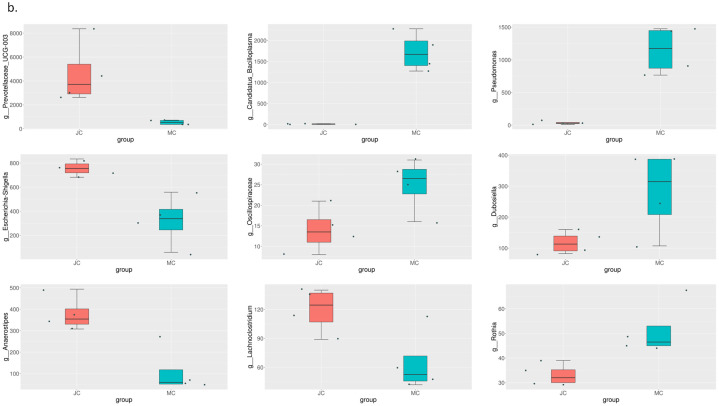
MetaStat analysis results at the order classification level. Differences between the MC group and the JC group.

**Fig 8:**
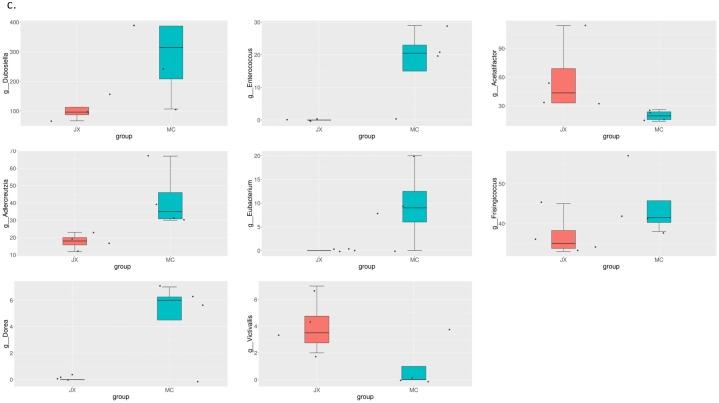
MetaStat analysis results at the order classification level. Differences between the MC group and the JX group.

Comparing the rats in the model group and the rats in the sham-operated group in the second month, we found significant differences in the abundance of Firmicutes, Bacteroidota, Campilobacterota, Cyanobacteria, Actinobacteria, and Desulfobacterota at the phylum level. In the third month, only Proteobacteria and Elusimicrobiota had significant differences. The difference between the model group and the estrogen group was smaller than that between the model group and the sham-operated group. The results are shown in Fig 6–8.

## 4 Discussion

Population aging is a major challenge for hospice care worldwide. As the population aging process intensifies, osteoporosis is becoming an increasingly major burden on global healthcare [20]. Studies have shown that about 1/3 of women and 1/5 of men in Western societies will suffer from osteoporotic fractures after the age of 50 [21]. The situation is even worse in East Asian countries [22,23]. With the deepening of research, gut microbiome dysbiosis has been considered an indispensable and important link in the process of estrogen deficiency leading to osteoporosis [11,24,25]. In this investigation, we established a rat model of postmenopausal osteoporosis through OVX surgery. Within 3 months post-surgery, we monitored alterations in serum estrogen, TNF-α, BMD, bone tissue morphology, and intestinal microbiota diversity. Our findings indicate that OVX surgery led to a progressive decline in serum estrogen levels and an imbalance in gut microbiota. This imbalance led to an increase in serum inflammatory immune factors, which in turn led to bone loss and PMOP. Estrogen therapy could improve this to some extent, which is consistent with previous studies [26,27].

After menopause, estrogen deficiency does not immediately cause osteoporosis. Bone loss is usually gradual and progressive, and it goes through two stages: a rapid loss in the first 5 years before menopause and a more gradual loss in the 10 years after menopause [28–29]. Estrogen plays a critical role in maintaining bone health by promoting bone formation and inhibiting bone resorption. Postmenopausal estrogen deficiency is the main mechanism of PMOP [30]. Estrogen deficiency can increase the levels of immune factors, which can interfere with bone metabolism through mechanisms such as the OPG/RANKL pathway. This can lead to bone metabolism imbalance, bone loss, and eventually the development of postmenopausal osteoporosis [8,31,32]. The data from this study revealed a progressive decline in estrogen levels and a concomitant increase in inflammatory factors following ovariectomy. Additionally, the rats exhibited a gradual reduction in bone density, with significant loss observed within the first month and a slower rate of bone loss over the subsequent two months, and this situation is more closely aligned with the human condition. Treatment with estrogen effectively inhibited both bone loss and the elevation of inflammatory factors in the rats.

On the other hand, estrogen deficiency can also affect intestinal mucosal barrier permeability, leading to bacterial translocation and altered gut microbiota diversity, this can then affect various bone properties by modulating the gut-bone axis [30,33,34]. The gut mucosal barrier is a physical and biochemical barrier that lines the gut wall and protects the body from harmful substances and microorganisms. It is also essential for maintaining a healthy balance of gut microbiota [35]. Damage to the intestinal mucosal barrier can lead to bacterial translocation and microbial imbalance, which can cause local long-term inflammatory responses in the gut. These inflammatory responses can then affect the levels of immune factors in the blood throughout the body [36]. At the same time, pathogens from the gut are more likely to cross the intestinal barrier and enter the body, causing systemic inflammatory responses [37]. The results of 16s rRNA sequencing in this study revealed that Campylobacter, Actinomycetes, and Cyanobacteria may play a role in the mechanism of OVX-induced intestinal microbial imbalance, which has been previously reported to impact bone or bone cells. Furthermore, we observed that the microbial imbalance was partially corrected after OVX surgery, but not completely restored to the level of rats that did not undergo OVX.

In this study, we observed significant changes in the abundance of intestinal flora in rats during the second month following ovariectomy, consistent with previous research. However, by the third month, there was no significant difference in intestinal flora abundance in the OVX group, possibly indicating a partial restoration of intestinal microecology over time. Furthermore, our findings revealed that firmicutes and Bacteroides remained dominant across all rat groups, and the trend of decreasing and then increasing levels of firmicutes in OVX rats differed from those in the sham group, whose levels remained constant. Even by the third-month, firmicutes levels in OVX rats did not show a clear increase compared to the sham group, which was different from previous studies. This difference likely reflects the establishment of a new equilibrium in the rats’ Gut Microbiota by the third month post-surgery, indicating a partial return toward normalcy. However, given the small sample size, the potential influence of inter-individual variation among the rats cannot be excluded. Increasing the sample size would help clarify this point.

In addition, gut microbes can also affect bone metabolism through their metabolites short-chain fatty acids (SCFAs) [38,39]. Short-chain fatty acids (SCFAs) constitute a primary group of metabolites generated through the metabolism of dietary fiber by intestinal microorganisms. These compounds serve various vital roles, including energy provisioning, regulation of intestinal peristalsis, preservation of the intestinal mucosal barrier, and modulation of metabolism [40]. Studies have shown that SCFAs may interfere with bone metabolism by affecting bone cell activity, regulating inflammatory response, and affecting the absorption of calcium and minerals in the intestine. And then, this has an impact on bone health [39,41]. Exploring the relationship between gut microbiota and bone health from the perspective of SCFAs also is one of the future directions of this research.

In general, this study aimed to explore the pathogenesis of PMOP from the perspective of intestinal microorganisms. The changing trend of various indicators in rats after OVX was analyzed using 16s rRNA sequencing and other methods within a 3-month period. However, due to funding and other limitations, this study has some limitations. For example, we did not discuss the trend of changes in the levels of intestinal microbial metabolites SCFAs. It is also difficult to explore the relationship between intestinal microbial imbalance and bone loss from the perspective of the “gut-bone axis”. Furthermore, This study was relatively small, and further studies with larger sample sizes are needed. Our future studies should explore the relationship between estrogen deficiency-induced intestinal mucosal barrier damage, levels of gut microbial metabolites, specific bacterial species, and bone loss. These findings will help to further understand the pathogenesis of PMOP.

## 5 Conclusions

In summary, the data reveals the changing trends of estradiol, TNF-α, and gut microbiota in the pathogenesis of PMOP. Estrogen deficiency results in a gradual increase in the levels of inflammatory markers in the bloodstream, resulting in increased bone resorption. In contrast, disruption of the intestinal microbiota peaked in the second month and then gradually returned to a relatively balanced state. Despite estrogen intervention, the gut microbiota of the rat could not fully return to its normal state. These findings still need to be verified by further animal experiments.

## References

[1] Li X, Wu K, Dong Q, Chen H, Li C, Ren Z, et al. Overall adjustment acupuncture improves osteoporosis and exerts an endocrine-modulating effect in ovariectomized rats. Front Endocrinol. 2022;13:1074516. doi: 10.3389/fendo.2022.1074516 PMC9712736 36465626

[2] Yuan W, Yang M, Zhu Y. Development and validation of a gene signature predicting the risk of postmenopausal osteoporosis. Bone Joint Res. 2022;11(8):548–60. doi: 10.1302/2046-3758.118.bjr-2021-0565.r1 35920104 PMC9396926

[3] Carey JJ, Chih-Hsing Wu P, Bergin D. Risk assessment tools for osteoporosis and fractures in 2022. Best Pract Res Clin Rheumatol. 2022;36(3):101775. doi: 10.1016/j.berh.2022.101775 36050210

[4] Rozenberg S, Al-Daghri N, Aubertin-Leheudre M, Brandi M-L, Cano A, Collins P, et al. Is there a role for menopausal hormone therapy in the management of postmenopausal osteoporosis? Osteoporos Int. 2020;31(12):2271–86. doi: 10.1007/s00198-020-05497-8 32642851 PMC7661391

[5] Ongphiphadhanakul B, Chanprasertyothin S, Chailurkit L, Chansirikarn S, Puavilai G, Rajatanavin R. Differential associations of residual estradiol levels with bone mineral density and serum lipids in postmenopausal women with osteoporosis. Maturitas. 2004;48(3):193–6. doi: 10.1016/j.maturitas.2003.08.007 15207884

[6] Fang H, Zhang H, Wang Z, Zhou Z, Li Y, Lu L. Systemic immune-inflammation index acts as a novel diagnostic biomarker for postmenopausal osteoporosis and could predict the risk of osteoporotic fracture. J Clin Lab Anal. 2020;34(1):e23016. doi: 10.1002/jcla.23016 31423643 PMC6977145

[7] Bhatnagar A, Kekatpure AL. Postmenopausal Osteoporosis: A Literature Review. Cureus. 2022;14(9):e29367. doi: 10.7759/cureus.29367 36299953 PMC9586717

[8] Fischer V, Haffner-Luntzer M. Interaction between bone and immune cells: Implications for postmenopausal osteoporosis. Semin Cell Dev Biol. 2022;123:14–21. doi: 10.1016/j.semcdb.2021.05.014 34024716

[9] Lisco G, Triggiani D, Giagulli VA, De Pergola G, Guastamacchia E, Piazzolla G, et al. Endocrine, Metabolic, and Immune Pathogenesis of Postmenopausal Osteoporosis. Is there a Therapeutic Role in Natural Products? Endocr Metab Immune Disord Drug Targets. 2023;23(10):1278–90. doi: 10.2174/1871530323666230330121301 37005529

[10] Yang X, Chang T, Yuan Q, Wei W, Wang P, Song X, et al. Changes in the composition of gut and vaginal microbiota in patients with postmenopausal osteoporosis. Front Immunol. 2022;13. doi: 10.3389/fimmu.2022.930244 PMC9411790 36032115

[11] Wang H, Liu J, Wu Z, Zhao Y, Cao M, Shi B, et al. Gut microbiota signatures and fecal metabolites in postmenopausal women with osteoporosis. Gut Pathog. 2023;15(1). doi: 10.1186/s13099-023-00553-0 PMC10324172 37415173

[12] Wang J, Wang Y, Gao W, Wang B, Zhao H, Zeng Y, et al. Diversity analysis of gut microbiota in osteoporosis and osteopenia patients. PeerJ. 2017;5:e3450. doi: 10.7717/peerj.3450 PMC5474093 28630804

[13] Guo M, Liu H, Yu Y, Zhu X, Xie H, Wei C, et al. Lactobacillus rhamnosus GG ameliorates osteoporosis in ovariectomized rats by regulating the Th17/Treg balance and gut microbiota structure. Gut Microbes. 2023;15(1):2190304. doi: 10.1080/19490976.2023.2190304 36941563 PMC10038048

[14] Li J-Y, Chassaing B, Tyagi AM, Vaccaro C, Luo T, Adams J, et al. Sex steroid deficiency-associated bone loss is microbiota dependent and prevented by probiotics. J Clin Invest. 2016;126(6):2049–63. doi: 10.1172/JCI86062 27111232 PMC4887186

[15] Liu F-X, Tan F, Fan Q-L, Tong W-W, Teng Z-L, Ye S-M, et al. Zuogui Wan improves trabecular bone microarchitecture in ovariectomy-induced osteoporosis rats by regulating orexin-A and orexin receptor. J Tradit Chin Med. 2021;41(6):927–34. doi: 10.19852/j.cnki.jtcm.20210903.001 34939389

[16] Magoč T, Salzberg SL. FLASH: fast length adjustment of short reads to improve genome assemblies. Bioinformatics. 2011;27(21):2957–63. doi: 10.1093/bioinformatics/btr507 21903629 PMC3198573

[17] Bokulich NA, Subramanian S, Faith JJ, Gevers D, Gordon JI, Knight R, et al. Quality-filtering vastly improves diversity estimates from Illumina amplicon sequencing. Nat Methods. 2013;10(1):57–9. doi: 10.1038/nmeth.2276 23202435 PMC3531572

[18] Edgar RC, Haas BJ, Clemente JC, Quince C, Knight R. UCHIME improves sensitivity and speed of chimera detection. Bioinformatics. 2011;27(16):2194–200. doi: 10.1093/bioinformatics/btr381 21700674 PMC3150044

[19] Wang Y, Guo H, Gao X, Wang J. The Intratumor Microbiota Signatures Associate With Subtype, Tumor Stage, and Survival Status of Esophageal Carcinoma. Front Oncol. 2021;11. doi: 10.3389/fonc.2021.754788 PMC8578860 34778069

[20] Bone AE, Gomes B, Etkind SN, Verne J, Murtagh FEM, Evans CJ, et al. What is the impact of population ageing on the future provision of end-of-life care? Population-based projections of place of death. Palliat Med. 2018;32(2):329–36. doi: 10.1177/0269216317734435 29017018 PMC5788077

[21] Lorentzon M, Johansson H, Harvey NC, Liu E, Vandenput L, McCloskey EV, et al. Osteoporosis and fractures in women: the burden of disease. Climacteric. 2021;25(1):4–10. doi: 10.1080/13697137.2021.1951206 34319208

[22] Chen X, Giles J, Yao Y, Yip W, Meng Q, Berkman L, et al. The path to healthy ageing in China: a Peking University–Lancet Commission. The Lancet. 2022;400(10367):1967–2006. doi: 10.1016/s0140-6736(22)01546-x PMC9801271 36423650

[23] Ikegami N, Rice T. Controlling Spending For Health Care And Long-Term Care: Japan’s Experience With A Rapidly Aging Society. Health Aff (Millwood). 2023;42(6):804–12. doi: 10.1377/hlthaff.2022.00700 37276471

[24] Xu Q, Li D, Chen J, Yang J, Yan J, Xia Y, et al. Crosstalk between the gut microbiota and postmenopausal osteoporosis: Mechanisms and applications. Int Immunopharmacol. 2022;110:108998. doi: 10.1016/j.intimp.2022.108998 35785728

[25] Bizzoca D, Solarino G, Vicenti G, Moretti L, Nappi VS, Belluati A, et al. Novel directions in the study of osteoporosis: focus on gut microbiota as a potential therapeutic target. J Biol Regul Homeost Agents. 2020;34(4 Suppl. 3):29–35. Congress of the Italian Orthopaedic Research Society. 33261254

[26] Cao Z, Liu W, Bi B, Wu H, Cheng G, Zhao Z. Isoorientin ameliorates osteoporosis and oxidative stress in postmenopausal rats. Pharm Biol. 2022;60(1):2219–28. 10.1080/13880209.2022.2142614 36382865 PMC9673777

[27] Abdelfattah Abulfadle K, Refaat Abdelkader Atia R, Osama Mohammed H, Saad Ramadan R, Mohammed NA. The potential anti-osteoporotic effect of exercise-induced increased preptin level in ovariectomized rats. Anat Sci Int. 2023;98(1):22–35. doi: 10.1007/s12565-022-00666-7 35507276

[28] Zhai G, Hart DJ, Valdes AM, Kato BS, Richards JB, Hakim A, et al. Natural history and risk factors for bone loss in postmenopausal Caucasian women: a 15-year follow-up population-based study. Osteoporos Int. 2008;19(8):1211–7. doi: 10.1007/s00198-008-0562-x 18305885

[29] Ho-Pham LT, Nguyen HG, Nguyen-Pham SQ, Hoang DK, Tran TS, Nguyen TV. Longitudinal changes in bone mineral density during perimenopausal transition: the Vietnam Osteoporosis Study. Osteoporos Int. 2023;34(8):1381–7. doi: 10.1007/s00198-023-06757-z 37106043

[30] Chen C, Lei H, Zhao Y, Hou Y, Zheng H, Zhang C, et al. A novel small molecule effectively ameliorates estrogen deficiency-induced osteoporosis by targeting the gut-bone signaling axis. Eur J Pharmacol. 2023;954:175868. doi: 10.1016/j.ejphar.2023.175868 37369296

[31] Zhang W, Gao R, Rong X, Zhu S, Cui Y, Liu H, et al. Immunoporosis: Role of immune system in the pathophysiology of different types of osteoporosis. Front Endocrinol (Lausanne). 2022;13:965258. doi: 10.3389/fendo.2022.965258 36147571 PMC9487180

[32] Li H, Deng W, Qin Q, Lin Y, Liu T, Mo G, et al. Isoimperatorin attenuates bone loss by inhibiting the binding of RANKL to RANK. Biochem Pharmacol. 2023;211:115502. doi: 10.1016/j.bcp.2023.115502 36921635

[33] Jeyaraman M, Nallakumarasamy A, Jain VK. Gut Microbiome - Should we treat the gut and not the bones? J Clin Orthop Trauma. 2023;39:102149. doi: 10.1016/j.jcot.2023.102149 37009327 PMC10064415

[34] Ibrahim I, Syamala S, Ayariga JA, Xu J, Robertson BK, Meenakshisundaram S, et al. Modulatory Effect of Gut Microbiota on the Gut-Brain, Gut-Bone Axes, and the Impact of Cannabinoids. Metabolites. 2022;12(12):1247. doi: 10.3390/metabo12121247 36557285 PMC9781427

[35] Bao K, Wang M, Liu L, Zhang D, Jin C, Zhang J, et al. Jinhong decoction protects sepsis-associated acute lung injury by reducing intestinal bacterial translocation and improving gut microbial homeostasis. Front Pharmacol. 2023;14:1079482. doi: 10.3389/fphar.2023.1079482 37081964 PMC10110981

[36] Chen Y-C, Greenbaum J, Shen H, Deng H-W. Association Between Gut Microbiota and Bone Health: Potential Mechanisms and Prospective. J Clin Endocrinol Metab. 2017;102(10):3635–46. doi: 10.1210/jc.2017-00513 28973392 PMC5630250

[37] Sardinha-Silva A, Alves-Ferreira EVC, Grigg ME. Intestinal immune responses to commensal and pathogenic protozoa. Front Immunol. 2022;13:963723. doi: 10.3389/fimmu.2022.963723 36211380 PMC9533738

[38] He W, Xie Z, Thøgersen R, Rasmussen MK, Zachariassen LF, Jørgensen NR, et al. Effects of Calcium Source, Inulin, and Lactose on Gut-Bone Associations in an Ovarierectomized Rat Model. Mol Nutr Food Res. 2022;66(8):e2100883. doi: 10.1002/mnfr.202100883 35107857 PMC9287054

[39] Zaiss MM, Jones RM, Schett G, Pacifici R. The gut-bone axis: how bacterial metabolites bridge the distance. J Clin Invest. 2019;129(8):3018–28. doi: 10.1172/JCI128521 31305265 PMC6668676

[40] Lange O, Proczko-Stepaniak M, Mika A. Short-Chain Fatty Acids-A Product of the Microbiome and Its Participation in Two-Way Communication on the Microbiome-Host Mammal Line. Curr Obes Rep. 2023;12(2):108–26. doi: 10.1007/s13679-023-00503-6 37208544 PMC10250490

[41] Yan J, Herzog JW, Tsang K, Brennan CA, Bower MA, Garrett WS, et al. Gut microbiota induce IGF-1 and promote bone formation and growth. Proc Natl Acad Sci U S A. 2016;113(47):E7554–63. doi: 10.1073/pnas.1607235113 27821775 PMC5127374

